# Impact of COVID-19 pandemic on physical and mental health status and care of adults with epilepsy in Germany

**DOI:** 10.1186/s42466-022-00209-5

**Published:** 2022-09-22

**Authors:** Kimberly Körbel, Felix Rosenow, Margarita Maltseva, Heiko Müller, Juliane Schulz, Panagiota-Eleni Tsalouchidou, Lisa Langenbruch, Stjepana Kovac, Katja Menzler, Mario Hamacher, Felix von Podewils, Laurent M. Willems, Catrin Mann, Adam Strzelczyk

**Affiliations:** 1 Epilepsy Center Frankfurt Rhine-Main, Center of Neurology and Neurosurgery, Goethe-University and University Hospital Frankfurt, Schleusenweg 2-16 (Haus 95), 60528 Frankfurt am Main, Germany; 2 LOEWE Center for Personalized Translational Epilepsy Research (CEPTeR), Goethe-University Frankfurt, Frankfurt am Main, Germany; 3 HA Hessen Agentur GmbH, Wiesbaden, Germany; 4 Department of Neurology, University Hospital Greifswald, Greifswald, Germany; 5 Epilepsy Center Hessen and Department of Neurology, Philipps-University Marburg, Marburg (Lahn), Germany; 6 Epilepsy Center Münster-Osnabrück, Department of Neurology with Institute of Translational Neurology, Westfälische Wilhelms-University, Münster, Germany; 7 Department of Neurology, Klinikum Osnabrück, Osnabrück, Germany

**Keywords:** Epilepsy, Seizure, COVID-19, SARS-CoV-2, Disease burden, Antiseizure medication

## Abstract

**Background:**

To mitigate the potential consequences of the coronavirus disease 2019 (COVID-19) pandemic on public life, the German Federal Government and Ministry of Health enacted a strict lockdown protocol on March 16, 2020. This study aimed to evaluate the impact of the COVID-19 pandemic on physical and mental health status and the supply of medical care and medications for people with epilepsy (PWE) in Germany.

**Methods:**

The Epi2020 study was a large, multicenter study focused on different healthcare aspects of adults with epilepsy. In addition to clinical and demographic characteristics, patients were asked to answer a questionnaire on the impact of the first wave of the COVID-19 pandemic between March and May 2020. Furthermore, the population-based number of epilepsy-related admissions in Hessen was evaluated for the January-June periods of 2017–2020 to detect pandemic-related changes.

**Results:**

During the first wave of the pandemic, 41.6% of PWE reported a negative impact on their mental health, while only a minority reported worsening of their seizure situation. Mental and physical health were significantly more negatively affected in women than men with epilepsy and in PWE without regular employment. Moreover, difficulties in ensuring the supply of sanitary products (25.8%) and antiseizure medications (ASMs; 19.9%) affected PWE during the first lockdown; no significant difference regarding these impacts between men and women or between people with and without employment was observed. The number of epilepsy-related admissions decreased significantly during the first wave.

**Conclusions:**

This analysis provides an overview of the general and medical care of epilepsy patients during the COVID-19 pandemic. PWE in our cohort frequently reported psychosocial distress during the first wave of the pandemic, with significant adverse effects on mental and physical health. Women and people without permanent jobs especially reported distress due to the pandemic. The COVID‐19 pandemic has added to the mental health burden and barriers to accessing medication and medical services, as self-reported by patients and verified in population-based data on hospital admissions.

***Trial registration*:**

German Clinical Trials Register (DRKS), DRKS00022024. Registered October 2, 2020, http://www.drks.de/DRKS00022024

## Background

In the first quarter of 2020, the severe acute respiratory syndrome (SARS) coronavirus 2 (SARS-CoV-2) and its clinical manifestation, coronavirus disease 2019 (COVID-19), reached Germany as the pandemic spread globally [1, 2]. To mitigate the potential consequences of the pandemic on public life, the German Federal Government and Ministry of Health enacted a strict lockdown protocol beginning on March 16, 2020 [3]. As a central measure, a shutdown of elective procedures in hospitals and a reduction of outpatient and inpatient services were imposed to provide sufficient intensive care capacity and protect the healthcare system from being overwhelmed. The German healthcare system is based on a broad supply of general practitioners as the first line of patient care and specialist medical practitioners as a second line (e.g., neurologists in private practice) [4, 5]. In addition, there are specialized hospital outpatient clinics (e.g., specialized epilepsy centers) that serve as the primary resource for severely ill patients. As a consequence of the lockdown protocol, access to medical care for patients with epilepsy and other chronic diseases that require specialized inpatient or outpatient treatment was limited [6]. In addition to medical aspects, social distancing and a reduced supply of sanitary and grocery goods also adversely affected people’s mental state [7–9]. Moreover, a higher incidence of COVID-19 infection and increased fatality during hospitalization were reported for patients with active epilepsy [10].

This multicenter study aimed to investigate the impact of the global COVID-19 pandemic and the secondary restrictions imposed on PWE in Germany during its first wave.

## Materials and methods

### Study design

This investigation is based on data made available by the Epi2020 study, a large, multicenter, questionnaire-based study performed in late 2020 that was focused on different aspects of healthcare in adults with epilepsy in Germany [11–15]. PWE were recruited in outpatient care at four epilepsy centers that provide a full range of neurological care with expertise in epileptology and intensive care medicine. While the Epilepsy Center Frankfurt Rhine-Main has a primarily urban catchment area of the city of Frankfurt am Main (population: 764,104, year 2020; www.statistik-hessen.de), the epilepsy centers in Greifswald (59,282, year 2020; www.statistik-mv.de), Marburg (population: 76,401, year 2020; www.statistik-hessen.de) and Münster (316,403, year 2020; www.it.nrw) provide care as the only neurologic departments in their cities and surrounding rural areas, with care for populations of more than half a million each [16]. Due to its representative population structure, the area around Marburg was used earlier for a population-based estimate of the incidence of SE in Germany [16]. This study was approved by the ethics committee of Goethe-University Frankfurt (reference number 19-440) and registered with the German Clinical Trials Register (https://www.drks.de/DRKS00022024; Universal Trial Number: U1111-1252-5331). We adhered to the Strengthening the Reporting of Observational Studies in Epidemiology (STROBE) guidelines [17].

Inclusion criteria were the confirmed diagnosis of epilepsy in line with the recommendations of the International League Against Epilepsy (ILAE) [18, 19] and a minimum age of 18. Participation required written consent from the participants themselves. Patients with an uncertain epilepsy diagnosis were excluded, as well as patients with a language barrier. The Epi2020 questionnaire was completed either by the patients alone or in cases of mild intellectual or physical disability with the assistance of their family members. The questionnaire was completed between October and December 2020. Patients were asked about the disease- and care-related impacts of the COVID-19 pandemic during its first wave (March 2020 to May 2020). Nine patients were excluded from the final analysis due to missing answers to questions pertinent to this study. The impact of the pandemic on seizure frequency, their general epilepsy situation (as a clinical global impression reported by the patients themselves, similar to the clinical global impression scale (CGI) [20]) and physical and mental well-being were assessed using a 7-item Likert scale. A 4-item Likert scale was used to evaluate to what extent subjects felt threatened by the pandemic and to what extent they were restricted in their working lives. In addition to the diagnosis and course of COVID-19 infection during the first wave, the impact of supply problems involving medical care, ASMs, food, and sanitary products was evaluated. Patients were encouraged to report any problems and difficulties they experienced during the lockdown in free text contributions. In addition, information concerning the etiology, severity, and duration of epilepsy, current ASM treatment, and sociodemographic data was collected. Seizure freedom was defined as complete seizure control for > 1 year at the time of the study.

To evaluate the impact of the COVID-19 pandemic on patient care in the catchment area of two recruiting epilepsy centers (Frankfurt and Marburg), population-based weekly numbers for epilepsy-related admissions at clinics in the state of Hessen, Germany, were recorded (identified by ICD-10 codes G40.x and G41.x; data provided by HA Hessen Agentur GmbH, Wiesbaden, Germany). The RITS-Toolbox (Kaust Biostatistics Research Group [21, 22]) was used to perform an interrupted time-series analysis (ITSA) on admission numbers for the period between January and June 2020 and the mean admission numbers for the periods between January and June in the years 2017 to 2019. Within ITSA, the most statistically significant change point in a data series is identified prior to determining whether it deviates significantly from the rest of the series. The ITSA results were visualized using simple linear regression in the pre- and post-changepoint period with GraphPad Prism (GraphPad Software, San Diego, CA, USA) and Pixelmator Pro (Pixelmator, Vilnius, Lithuania).

### Statistical comparison

Data were analyzed using SPSS version 27 (IBM Corporation, Armonk, NY, USA). Descriptive data are presented as median, mean ± standard deviation (SD), minimum, maximum, and percentage values. Differences in worsening mental and physical health, seizure control, and access to supplies between patient groups were evaluated using the Mann–Whitney U test (*U*) in cases of ordinal or non-normally distributed data. Spearman’s rank correlation (*r*_*s*_) was used to examine whether worsening mental and physical health, seizure control, or supplies were associated with patient age, number of ASMs, or seizure frequency. Two-sided *P*-values < 0.05 were considered significant in all statistical analyses.

## Results

### Patient cohort

A total of 477 PWE from four epilepsy centers were included in this study. Seizure-free status for at least one year was reported by 190 patients (39.8%). Information on sociodemographic, clinical, and epilepsy-related characteristics of the study population is presented in Table 1.

**Table 1:** Clinical and demographic characteristics of all patients (n = 477)

		Total n = 477
Mean age (median, range, SD)		40.3 (38, 18–83, 15.4)
Sex	Female	58.1% (277)
	Male	41.9% (200)
Seizure control	Seizure free > 1 year	39.8% (190)
	Persisting seizures	58.5% (279)
	n.a	1.7% (8)
Epilepsy type	Focal	67.9% (324)
	Idiopathic (genetic) generalized	21.0% (100)
	Unclassified	11.1% (53)
Mean number of ASMs (median, range, SD)		1.8 (2, 0–6, 0.95)
Employment status	Working	51.2% (244)
	Not working	34.1% (163)
	In training	9.0% (43)
	Other	3.8% (18)
	n.a	1.9% (9)
Living situation	Alone	26.2% (125)
	With partner or relatives	72.1% (344)
	n.a	1.7% (8)

*SD* Standard deviation, *ASMs* Antiseizure medications, *n.a.* No answer

Between March and May 2020, the COVID-19 infection status of 193 patients (40.9%) was determined with a polymerase chain reaction (PCR) assay. During the first wave, eight patients (1.7%) reported acute COVID-19 infections; one additional patient reported a positive PCR test. The affected patients reported multiple symptoms: general weakness (87.5%), sore throat (75%), headache (75%), cough (62.5%), loss of smell or taste (62.5%), abdominal pain (50%), and fever (37.5%). Their other symptoms included rhinorrhea (37.5%), aching limbs (37.5%), dyspnea (25%), diarrhea (25%), loss of appetite (25%), nausea (12.5%), and weight loss (12.5%). Two patients required inpatient treatment for their infection; no patients required ventilation. The seizure frequency remained stable for five patients with acute COVID-19 infection. One patient reported a large increase in seizure frequency, and one reported a small increase.

### Impact on physical and mental health

**Fig. 1:**
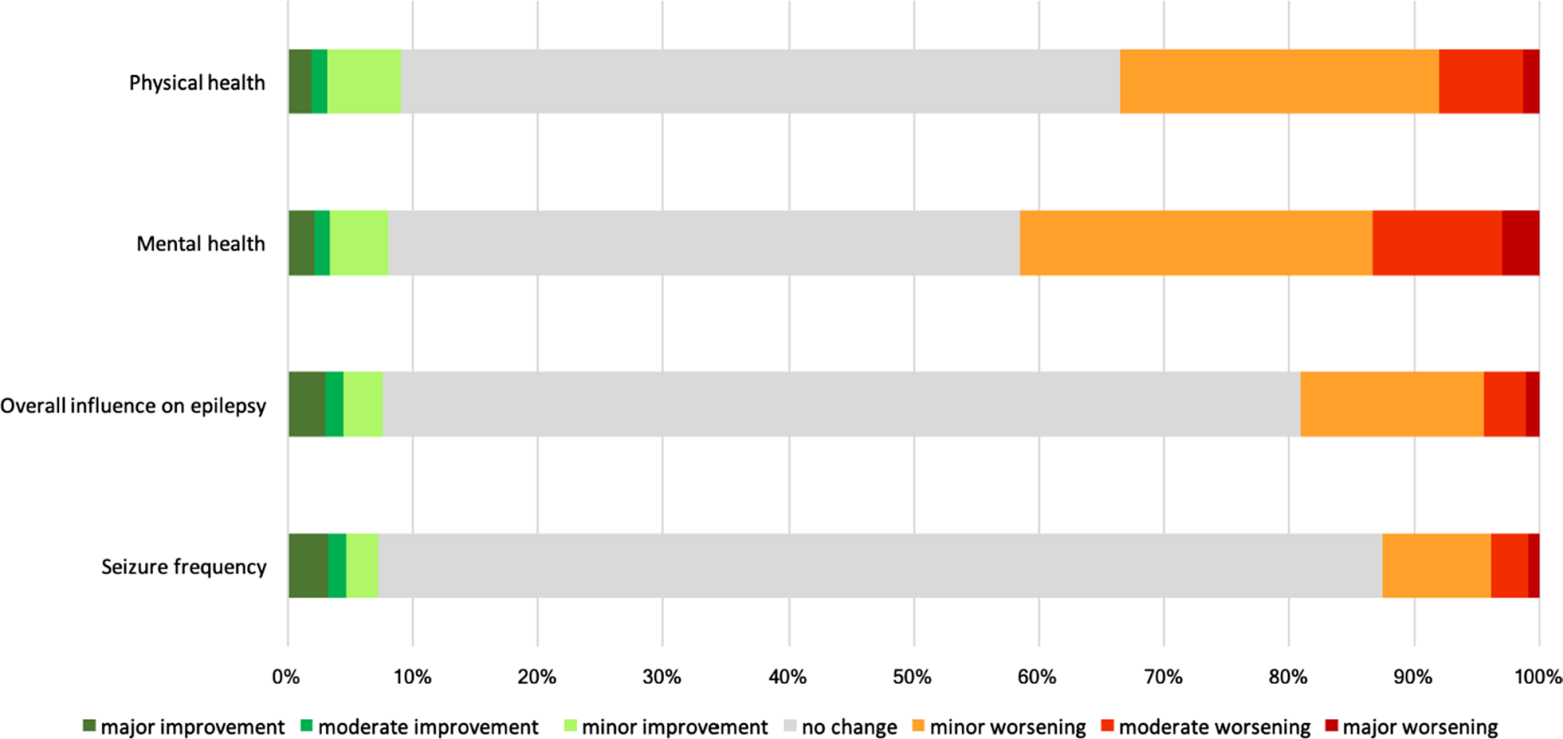
Effect of the first wave of the COVID-19 pandemic on physical health, mental health, the overall course of epilepsy, and seizure frequency, as reported on a 7-point Likert scale

PWE ratings on the impact of the COVID-19 pandemic and its associated restrictions in Germany on their physical and mental health are presented in Fig. 1. Some worsening of mental health was reported by 41.6% (n = 197) of PWE. A worsening in physical health was reported by one-third (33.5%, n = 159). The pandemic had a negative impact on general epilepsy disease status in 19.1% (n = 90). In particular, increased seizure frequency was reported by 12.5%. More than three-quarters felt generally threatened by the pandemic (n = 362, 76.2%).

### Impact on employment situation and supply

**Fig. 2:**
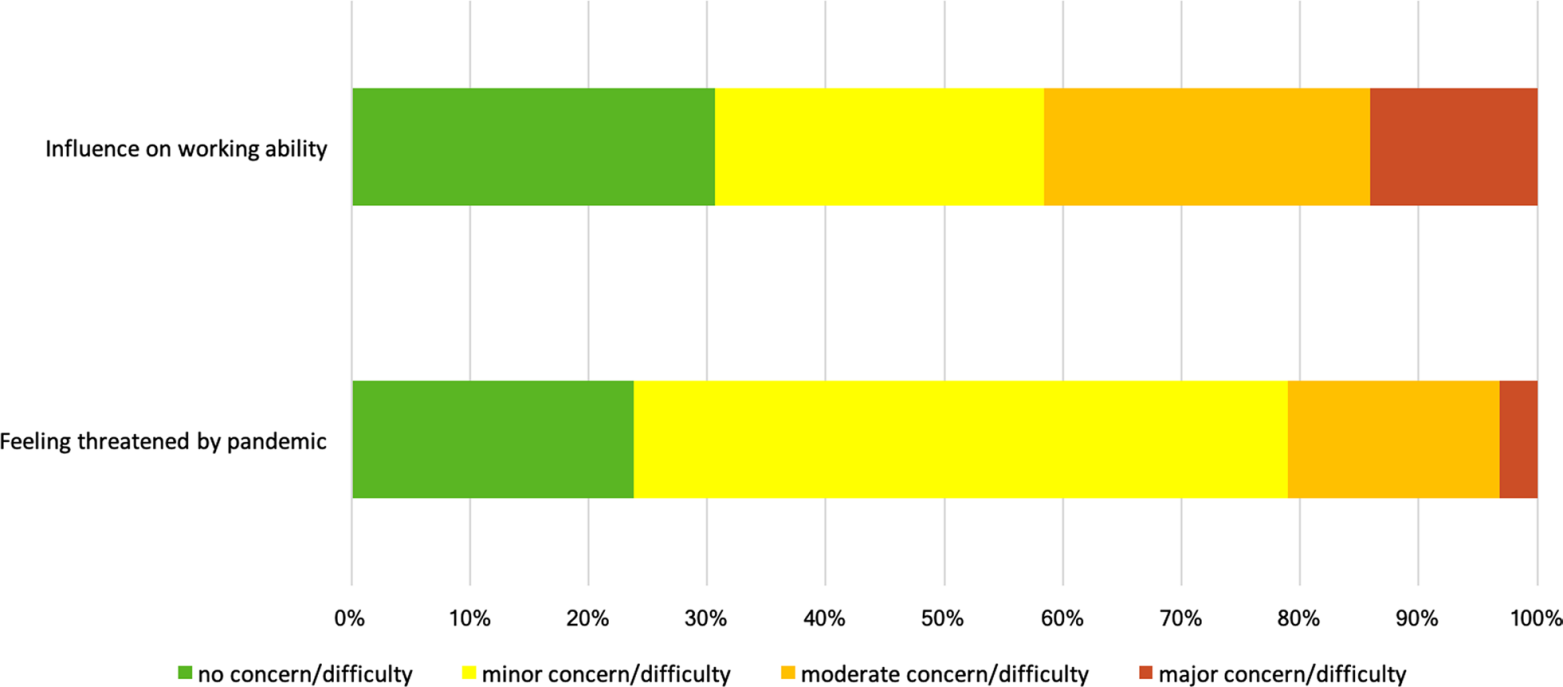
Effect of the first wave of the COVID-19 pandemic on the ability to work and feelings of being threatened by the pandemic, as reported on a 4-point Likert scale

The COVID-19 pandemic and its associated restrictions affected the working ability of PWE, for details please refer to Fig. 2. Only 52.5% of PWE in the cohort were employed. Concerns regarding their ability to work were reported by 69.4%, while 29.7% reported work interruptions between March and May 2020.

**Fig. 3:**
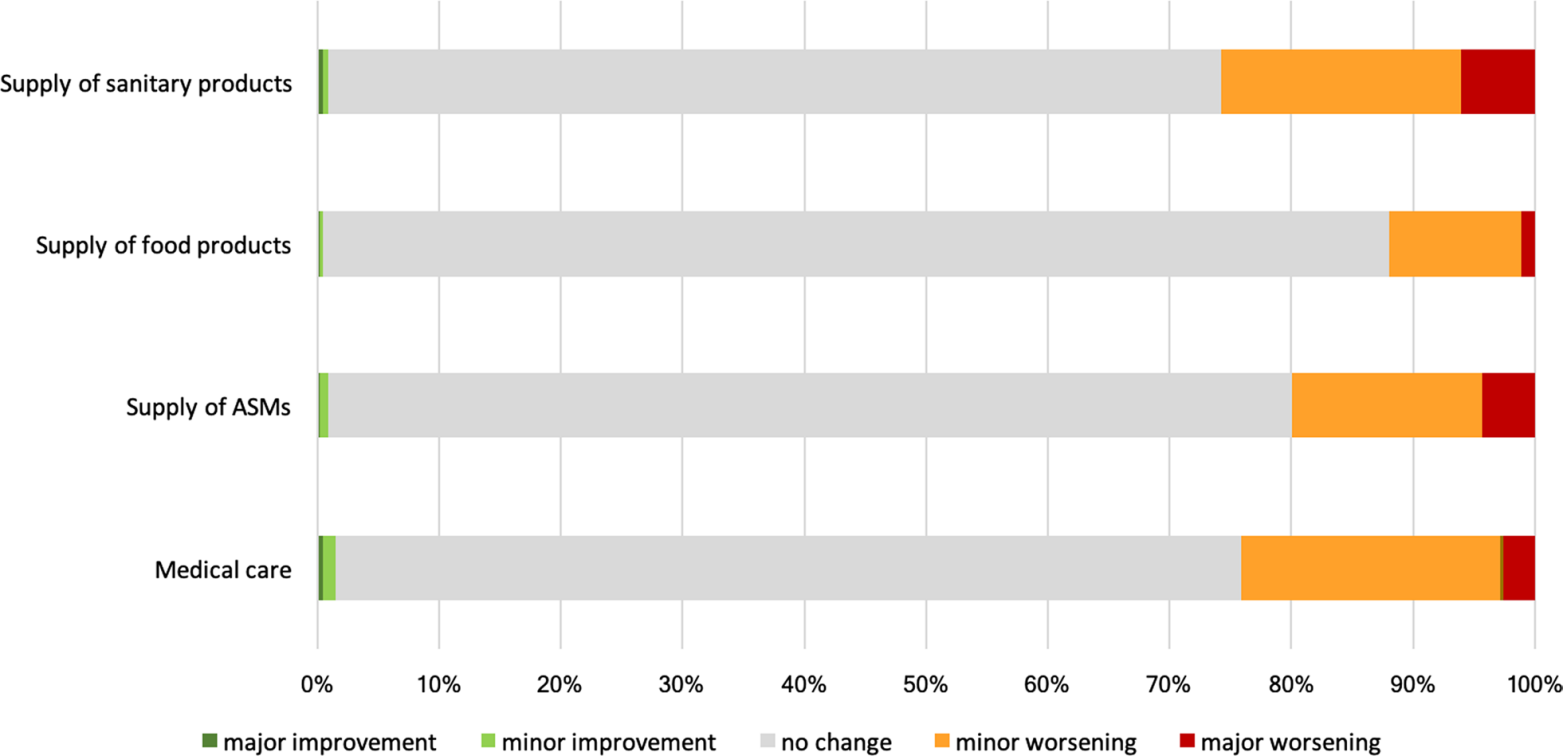
Effect of the first wave of the COVID-19 pandemic on the supply of sanitary products, food products, ASMs, and general medical care, as reported on a 5-point Likert scale

Particularly during the first wave of the COVID-19 pandemic, people faced limitations on the supply of various products, including medications and daily necessities, for details please refer to Fig. 3. More than a quarter of PWE (25.8%) reported difficulties accessing sanitary products. Additionally, 19.9% reported difficulties accessing ASMs, while 12% reported a restricted supply of food products between March and May 2020. Furthermore, 24% reported reduced access to routine medical care during the pandemic.

### Univariate analysis of effects and sociodemographics

During the first wave of the COVID-19 pandemic, employed PWE were significantly less likely to report a negative impact on their seizure situation (*p* = 0.016) or their epilepsy in general (*p* = 0.003) than PWE without employment. Employed PWE were also significantly less likely to report worsening in psychological well-being (*p* = 0.008) and were understandably more likely to have their employment or work activities disrupted by the pandemic (*p* < 0.001). The impact of the supply situation was not significantly different between employed and unemployed groups.

While the impact of the COVID-19 pandemic on seizure control in women with epilepsy was not significantly different from men with epilepsy, they were significantly more likely to report a worsening of their epilepsy in general (23% vs. 13.6%, *p* = 0.008). Additionally, mental and physical health were significantly more adversely affected in women than in men; 48.4% of women reported experiencing a worsening of their mental health, compared with 32.1% of men (*p* < 0.001). Moreover, a negative impact on physical health was reported by significantly more women (39.2%) than men (30%; *p* < 0.001). Furthermore, 80.1% of women and 71.0% of men described feeling threatened by the pandemic (*p* = 0.001). The reported impact on working life (*p* = 0.869) and the supply situation (supply of ASMs, *p* = 0.867; supply of food products, *p* = 0.236; supply of sanitary products, *p* = 0.903) were not significantly different between men and women with epilepsy.

Psychological and physical well-being, care situation, and work-life during the first wave of the COVID-19 pandemic were found not to differ significantly between PWE living with partners or family and PWE living alone. Moreover, seizure control was found to have no significant effect on reported concerns, health, or supply status.

There was no significant association between patient age, mental and physical well-being, seizure control, and feelings of being threatened during the first wave of the COVID-19 pandemic. However, patient age was negatively correlated with employment constraints (*r*_*s*_ = -0.260, *p* < 0.001). Moreover, number of ASMs taken was positively correlated with worsening of seizure control (*r*_*s*_ = 0.129, *p* = *0.005*), general worsening of epilepsy (*r*_*s*_ = 0.200, *p* < 0.001), and worsening of mental well-being (*r*_*s*_ = 0.105, *p* = 0.022). Experiencing problems with drug supply during the first wave was not correlated with the number of ASMs taken.

**Fig. 4:**
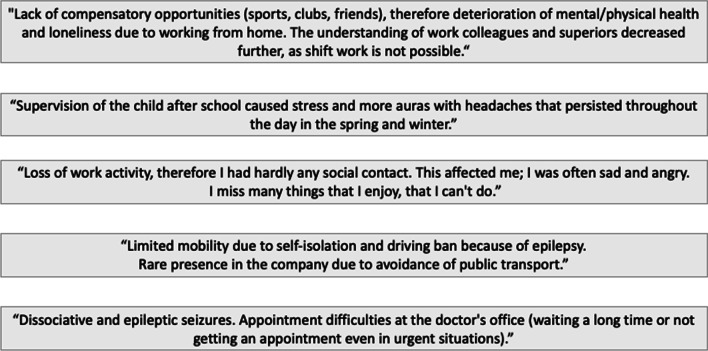
Examples of free-text responses and reactions to the first wave of the COVID-19 pandemic reported by PWE

In total, 161 PWE reported problems regarding the first wave of the pandemic and COVID-19 disease in free-text form. The most common answer was the loss of social contacts, which was often reported together with impacts on their work-life or the schooling of their children or themselves (e.g., the change to online classes or working from home). In addition, physical and mental health problems were frequently mentioned, such as those discussed above, followed by restrictions in sports or leisure activities. Moreover, the patients reported difficulties accessing general medical care, particularly inpatient procedures and outpatient appointments, and problems in their family life, including difficulty organizing childcare and conflicts with, or health issues of, family members. Example quotes from patient free-text responses are presented in Fig. 4, while Table 2 provides the number of issues mentioned, sorted into categories.

**Table 2:** Restrictions in the first wave of the COVID-19 pandemic, as reported in free-text responses by PWE

Restrictions in the first wave of the COVID-19 pandemic	*n* = 161 (100%)
Loss of social contacts	35 (21.7%)
Influence on work or school	30 (18.6%)
Mental health disorders, anxiety	25 (15.1%)
Physical health problems	21 (13.0%)
Restrictions on sports or hobbies	18 (11.2%)
Influence on medical care or appointments	15 (9.3%)
Problems in family	14 (8.7%)
Financial problems	9 (5.6%)
Problems caused by wearing a mask	8 (5.0%)
Mobility restrictions (e.g., public transport)	7 (4.3%)
Sleep disorders	5 (3.1%)
Side effects of medication	2 (1.2%)
Restrictions in the supply of:	
ASMs	10 (6.2%)
hygiene products	2 (1.2%)
food products	1 (0.6%)

Multiple answers were allowed

**Fig. 5:**
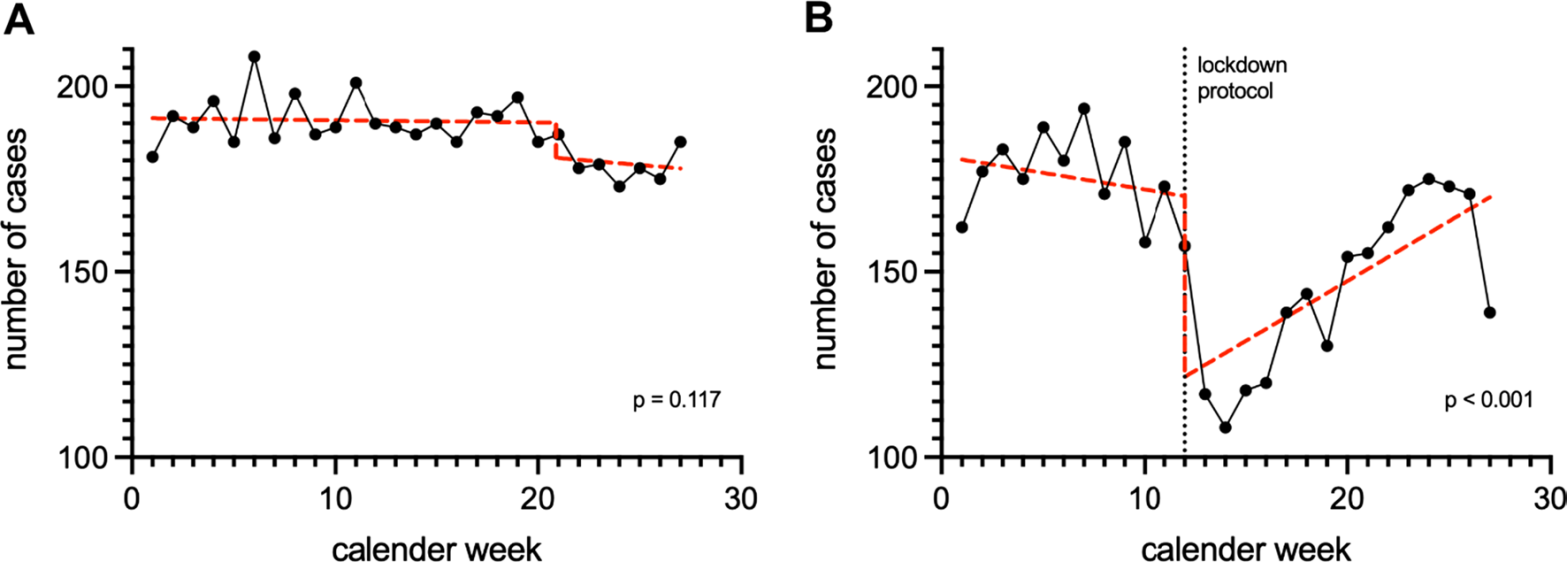
Population-based weekly admission numbers of patients to hospitals in the state of Hessen, Germany, due to epilepsy. **A**: January-June periods of 2017 to 2019; **B**: January-June period of 2020; Red line: simple linear regression for the pre- and post-change-point period

ITSA detected no significant change-point in averaged hospital admission numbers during the periods January–June in 2017–2019 (*p* = 0.117, Fig. 5A). However, it detected a significant trend deviation in the data series for January to June 2020, with a change-point in calendar week 12 (*p* < 0.001, Fig. 5B).

## Discussion

In this retrospective multicenter study, we explored the effects of the first wave of the COVID-19 pandemic on PWE in Germany. While only a minority of patients reported a worsening of their seizure situation during the first wave, more than 40% of PWE described a worsening of their mental state, where women were significantly more affected than men. In addition, more than 30% of patients reported a deterioration in physical condition.

The COVID-19 pandemic affected the care of patients around the world. Our results are in line with those of an internet-based, internationally distributed survey conducted by the ILAE during the first wave of the pandemic [23]. Among PWE respondents, 57.1% reported experiencing severe psychological distress, while 22.8% reported increased seizure frequency and difficulties in obtaining medications or accessing healthcare. These findings are consistent with earlier studies that found reduced hospital admission rates during the first lockdown in Germany and a negative impact on emergency procedures for neurological disorders (e.g., thrombectomy for acute ischemic stroke) [24, 25].

Our findings on the restricted supply of sanitary products and ASMs align with those of an earlier single-center study conducted at the start of the COVID-19 pandemic [6], as well as an online, multicountry internet survey that found 19.6% of PWE had difficulty obtaining ASMs, 50.4% screened positive for anxiety, and > 40% screened positive for depression [26], both referring to the same time period as our data. Moreover, both studies found depression and anxiety to be more common in females and PWE with financial problems, in agreement with our study. Importantly, ongoing psychological distress has also been reported by PWE during the later waves of the COVID-19 pandemic [27]. The mental distress similarly affected people with other chronical illnesses, e.g. arthritis or diabetes, which is referable to the loss of resources due to the pandemic [28, 29]. In addition to that, patients with physical and mental disabilities, like those suffering from developmental and epileptic encephalopathies, and their caregivers might be even more affected by the pandemic [30, 31].

Whether PWE are at increased risk of worsening seizures following SARS-CoV-2 infection remains to be determined. Because of the low incidence of COVID-19 in our cohort during the first wave of the pandemic, we were unable to draw reliable conclusions on this. Fear of experiencing more severe COVID-19 disease due to epilepsy was reported by 74.5% of PWE in Brazil, while dissatisfaction with current health status was reported by 36.7%, but related to the second pandemic wave in autumn 2020 [32]. In addition, fear of belonging to an “at-risk” group may contribute to increased psychological distress during the first wave of the pandemic. The available evidence, including our survey results, indicates that a large proportion of PWE experienced difficulties during the first wave of the pandemic. In addition, some PWE reported an overall increase in seizure frequency and difficulty accessing medical care, particularly medications, investigations, information, and self‐management. However, the respondents’ degree of psychological distress and pandemic-related loss of resources may have contributed to their increase in seizure frequency. Furthermore, efforts to avoid healthcare facilities and minimize unnecessary contact may have contributed to the barriers to medical care experienced by PWE.

Our data highlight how the COVID‐19 pandemic has added to the mental health burden of an already vulnerable group. There are increasing reports of barriers to obtaining advice from medical services and difficulty accessing medication throughout the pandemic [33].

Consensus guidance statements for professionals caring for PWE during the COVID-19 pandemic have been made available [34]. These recommendations focus on administering as much care as possible at home to keep PWE out of healthcare facilities where they will be at increased risk of COVID-19 exposure. They include strategies for rescue therapy and seizure risk minimization by ensuring a regular supply of medication.

High-risk individuals remain those with diseases that restrict mobility, respiratory conditions (including asthma), diabetes mellitus, hypertension, severe heart disease, impaired immune function due to underlying conditions or drug treatment, and older age, particularly when associated with frailty [35]. Current data suggest that PWE are no more likely to be infected with SARS-CoV-2, nor are they more likely to have severe manifestations of COVID-19 [36, 37]. The absence of association with severe COVID-19 disease is consistent with reports for other common chronic neurological disorders such as multiple sclerosis [38]. Moreover, epileptic seizure is rare as a symptom of acute or chronic COVID-19 infection [39, 40].

The COVID-19 pandemic has led to the redeployment of inpatient capacity to minimize strain on health systems worldwide, disrupting routine hospital services for all non-COVID patients. Overall inpatient admissions to German University hospitals decreased by 35% in weeks 1–4 and 30.3% in weeks 5 to 8 after the lockdown announcement in March 2020 compared to 2018 [25]. We have demonstrated a comparable decrease in PWE inpatient care with population-based data from Hessen, Germany.

Adherence of known PWE to ASM treatment appeared to remain stable during the lockdown in Germany. A recent study found reduced outpatient care during the first lockdown period for newly diagnosed PWE in Germany and fewer hospital admissions in PWE [41]. Nationally and internationally, video EEG monitoring units have been closed, or only specific emergencies have been treated [42]. Their prolonged closure resulted in a warning by the ILAE for the rapid resumption of this essential diagnostic service so as not to deny patients necessary treatment [43].

The COVID-19 pandemic has seen a pronounced dynamic fluctuation in infection numbers over the past two years, with evolving viral variants that have more recently shown increasing infectivity with decreasing disease severity. While global infection numbers were declining in April 2022, predicting future waves of the COVID-19 pandemic remains challenging [44].

To what extent the future pandemic situation and the “new normal” established in Germany will impact the psychological and physical situation of PWE remains the subject of further investigation. The results of our study are intended to sharpen our focus and understanding of this and other patient populations with chronic diseases to improve their care in future pandemics. In our opinion this could include a wider supply of telecommunication which showed a successful implementation for PWE in outpatient care in the first pandemic wave [6] or the use of an epilepsy electronic patient portal [45]. An important aim is creating a concept for maintaining the supply of medical products at early stages in future pandemic waves.

Despite its multicenter design, one limitation of our study is that it was conducted with PWE being treated at epilepsy centers in university hospitals, which implies the involvement of a select study cohort enriched for patients with focal and difficult-to-treat epilepsies.

## Conclusions

The first wave of the COVID-19 pandemic significantly impacted the mental and physical health of PWE in Germany and caused supply problems for medical, hygiene, and other goods. Women and people without permanent jobs reported the highest burden of distress from the pandemic. It has added to the mental health burden and barriers to accessing medical services and medications in an already vulnerable group. Our results suggest that in future pandemics or endemics, a focus on caring for patients with epilepsy and other chronic diseases is required to avoid exposing these groups to further stressors.

## Data Availability

The datasets used and analyzed during the current study are available from the corresponding author on reasonable request.

## Abbreviations

ASM: Antiseizure medication
CePTER: Center for personalized translational epilepsy research
CGI: Clinical global impression scale
COVID-19: Coronavirus Disease 2019
DRKS: Deutsches Register Klinischer Studien
ILAE: International League Against Epilepsy
ITSA: Interrupted time-series analysis
n.a.: No answer
p: P-value
PCR: Polymerase chain reaction
PWE: People with epilepsy
r_s_: Spearman’s rank correlation coefficient
SD: Standard deviation
STROBE: Strengthening the Reporting of Observational Studies in Epidemiology
vs: Versus
